# Variations in national availability of waivered buprenorphine prescribers by racial and ethnic composition of zip codes

**DOI:** 10.1186/s13011-022-00457-3

**Published:** 2022-05-25

**Authors:** Katherine A. Hirchak, Solmaz Amiri, Gordon Kordas, Oladunni Oluwoye, Abram J. Lyons, Kelsey Bajet, Judith A. Hahn, Michael G. McDonell, Aimee N. C. Campbell, Kamilla Venner

**Affiliations:** 1 Department of Community and Behavioral Health, Elson S. Floyd College of Medicine, Washington State University, PO Box 1495, Spokane, WA 99210-1495 USA; 2 Program of Excellence in Addictions Research, Washington State University, Spokane, WA USA; 3 Institute for Research and Education to Advance Community Health, Washington State University, Seattle, WA USA; 4 School of Social Policy and Practice, University of Pennsylvania, Philadelphia, PA USA; 5 University of California-San Francisco, San Francisco, CA USA; 6 Department of Psychiatry, Columbia University Irving Medical Center, New York, NY USA; 7 New York State Psychiatric Institute, New York, NY USA; 8 Center On Alcohol, Substance Use And Addictions, University of New Mexico, Albuquerque, NM USA; 9 Department of Psychology, University of New Mexico, Albuquerque, NM USA

**Keywords:** Black/African American, Hispanic/Latinx, American Indian and/or Alaska Native adult, Medications for opioid use disorder, Drug enforcement administration, Urban and rural

## Abstract

**Background:**

Opioid overdose remains a public health crisis in diverse communities. Between 2019 and 2020, there was an almost 40% increase in drug fatalities primarily due to opioid analogues of both stimulants and opioids. Medications for opioid use disorder (MOUD; e.g., buprenorphine) are effective, evidence-based treatments that can be delivered in office-based primary care settings. We investigated disparities in the proportion of national prescribers who have obtained a waiver issued to prescribe MOUD by demographic characteristics.

**Methods:**

Data for the secondary data analyses were obtained from the Drug Enforcement Administration that maintains data on waivered MOUD prescribers across the US. Proportion of waivered prescribers were examined by ZIP code, race and ethnicity composition, socioeconomic status, insurance, and urban–rural designation using generalized linear mixed effects models.

**Results:**

Compared with predominantly Non-Hispanic White ZIP codes, other racially and ethnically diverse areas had a higher proportion of waivered buprenorphine prescribers. Differences in prescriber availability between predominant racial group was dependent on rurality based on the interaction found in our fitted model. In metropolitan areas, we found that predominantly Non-Hispanic White ZIP codes had a lower rate of waivered prescribers compared to predominantly Black/African American ZIP codes.

**Conclusions:**

Our findings suggest that among AI/AN and Black/African American neighborhoods, availability of waivered prescribers may not be a primary barrier. However, availability of waivered prescribers and prescribing might potentially be an obstacle for Hispanic/Latinx and rural communities. Additional research to determine factors related to improving MOUD availability among diverse communities therefore remains vital to advancing health equity.

**Supplementary Information:**

The online version contains supplementary material available at 10.1186/s13011-022-00457-3.

## Introduction

The opioid overdose epidemic remains an urgent public health crisis across all racial and ethnic groups [1, 2]. In 2018, two million people over the age of 12 had an opioid use disorder diagnosis [3] and approximately 70% of all fatal drug overdoses in the United States were attributed to opioids [4]. The national discourse around the opioid epidemic has primarily focused on low-income rural non-Hispanic White (NHW) adults [5], due in part to national data revealing the highest rates of overdose among NHW adults [6]. However, this focus neglects underserved communities that have been severely impacted by opioid misuse related to recent increased prevalence of opioid use [7, 8]. For example, current research in the state of Washington has indicated that Black/African American [9], Hispanic/Latinx [10] and American Indian/Alaska Native (AI/AN) adults [10] experience higher rates of fatal and non-fatal opioid overdoses in both urban and rural areas as compared with NHW adults. Nationally this trend holds, with opioid related fatalities among Black/African American, Latinx/Hispanic and AI/AN adults rapidly increasing [11, 12]. Effective, appropriate, and accessible options to address opioid misuse are therefore essential for improving public health policy for all people.

Towards this goal, medication for opioid use disorder (MOUD; e.g., methadone, buprenorphine and extended release naltrexone) has been made more widely available to prevent overdose and support recovery. MOUD is the Food and Drug Administration’s approved evidence-based approach for the treatment of opioid use disorder [13–15]. The Drug Addiction Treatment Act (DATA) waiver allows eligible prescribers to receive training and register with the Drug Enforcement Administration to provide medications in office-based and primary care settings, places not typically viewed as treatment access points. Current eligible prescribers are physicians, physician assistants, nurse practitioners, clinical nurse specialists, certified nurse-midwives, and certified registered nurse anesthetists [13]. Depending on the waiver, a prescribers can treat up to 275 patients at a time [15]. Although these efforts have increased access for the treatment of opioid use disorder, there are barriers that continue to hinder access to care [16].

Socioeconomic and community characteristics have been shown to facilitate or impede access to receiving MOUD. For instance, type of insurance is associated with both increased (e.g., point of service insurance plan) and decreased access (e.g., Medicare) to treatment [17, 18]. Other neighborhood-level characteristics such as urban–rural designation and indicators of socioeconomic status (SES) can also differentially impact access to MOUD. A nationwide study found that small towns characterized by lower SES had lower mean access scores to office-based buprenorphine treatment [19]. Access to opioid treatment programs were also lower in micropolitan and small towns compared to metropolitan areas [19].

We build upon the existing research and use data from The Drug Enforcement Administration Registration Controlled Substances Act Information Database to examine individual and neighborhood level indicators previously associated with access to buprenorphine prescribers across the United States. To our knowledge, no study to date has used this dataset to examine potential differences in availability of waivered prescribers based upon the racial and ethnic composition of ZIP codes. This research is necessary to improve treatment access among underserved communities. To address this gap, we include race and ethnicity composition along with other population characteristics (i.e., urban/rural designation, insurance status, and SES characteristics) of ZIP codes to explore the association between geographic areas and number of buprenorphine waivered prescribers. This will help us better understand potential barriers and facilitators to treatment access across the United States.

## Methods

This ecological cross-sectional study was conducted at the ZIP code level [20]. Our dataset included 90% (28,993) of all ZIP codes in the U.S. We excluded ZIP codes with missing or zero population data. The Drug Enforcement Administration Registration Controlled Substances Act Information Database (formerly maintained by the National Technical Information Service) houses information for all prescribers across the United States that have ever had a waiver to prescribe MOUD. These data were requested by submitting a proposal describing this research, data storage, and data analysis. Once approval was obtained, the researchers were granted access to the online database that included data on type of business type (e.g., hospital), drug schedules, names and addresses of prescribers, and practitioner type. We downloaded the data which was current as of December 2020. Washington State Institutional Review Board determined that this study did not meet the criteria for human subjects research.

### Measures

#### Outcome variable

The outcome was the number of prescribers registered with the Drug Enforcement Administration Registration Controlled Substances Act Information Database per 100,00 population by ZIP code [21]. The addresses of the Drug Enforcement Administration (DEA) prescribers were geocoded using ESRI ArcGIS and R software. The number of prescribers were then calculated by each ZIP code.

#### Independent variables

Data on the independent variables were from the 2019 American Community Survey 5-year estimates [22, 23]. A categorical indicator of the predominant racial and ethnic composition for each ZIP code was developed in accordance with previously published research [24, 25]. We defined “predominantly” as greater than 50% of ZIP code residents identifying with one of the five racial or ethnic categories used in the Census: non-Hispanic (NH) AI/AN, NH Black, NH White (NHW), NH other races, or Hispanic/Latinx ethnicity regardless of race.

We used the 2019 American Community Survey data to calculate percentage of the population who are uninsured, on Medicare, or on Medicaid within each ZIP code. To examine SES characteristics, we used a validated composite score that assesses SES disadvantage, the area deprivation index. The area deprivation index includes 17 Census variables in 4 domains: housing, employment, education, and poverty [26, 27]. Index scores range from 1–100 with higher scores representing more deprivation [28]. We created a binary indicator of high deprivation (top 20%) as a proxy for low socioeconomic status [26, 29].

Block group rurality was defined using the Rural–Urban Commuting Area (RUCA) codes. RUCA codes categorize US Census tracts into rural and urban regions through measures of population data, urbanization and commuting information. Primary codes of 1–3 were classified as metropolitan; primary codes of 4–6 were classified as micropolitan; primary codes of 7–9 were classified as small towns; and primary codes of ten were classified as rural [30, 31]. We identified these variables based upon literature that reported factors that impact availability, access and delivery of MOUD at the individual and prescriber levels [17, 19, 32, 33].

### Statistical analysis

The number of neighborhoods were summarized by rurality and race/ethnicity via counts and percentages. Similarly, percentage uninsured, percentage on Medicare, and percentage on Medicaid were summarized with the interquartile ranges (minimum, maximum). The average number of DEA waivered prescribers per 100,000 residents was also summarized by rurality and race/ethnicity.

Differences in the number of DEA-waivered prescribers available were modeled using a generalized linear mixed effects model with a log link, assuming a negative binomial distribution. The final model included fixed effects for neighborhood predominant racial group, neighborhood rurality, an interaction between neighborhood predominant racial group and neighborhood rurality, SES (i.e., area deprivation index), proportion of the neighborhood insured, proportion of the neighborhood on Medicare, and proportion of neighborhood on Medicaid. Predominantly NHW, lowest SES (i.e., most-deprivation), and metropolitan RUCA designation were the reference categories for the independent variables. Also included in the model was a random intercept for state (to account for clustering) and a population offset (to account for the heterogeneity in ZIP code population sizes). Model estimates were placed on the per 100,000 resident scale for interpretability. To understand the interaction effect between racial group and rurality we performed pairwise comparisons between racial groups within each rurality type (10 contrasts within 4 rurality groups). *P*values were adjusted via the Tukey honestly significant difference test for multiple comparisons. To check for issues of multicollinearity we calculated the variance inflation factor (VIF) for each term in the fitted models. All analyses were performed in the R statistical software [34].

## Results

Across all rurality categories, the racial composition was classified as predominantly NHW, ranging from 80% in metropolitan areas to 94% in rural areas. Additionally, the overall median number of adults that were insured through Medicare was 5.5, the median number of adults on Medicaid was 12 and the median number of uninsured adults were 7. Fiftenn percent of the sample were categorized as most deprived ZIP codes. Complete characteristics by ZIP code can be found in Table 1.

**Table 1:** ZIP code characteristics

Characteristic	Overall, *N* = 28,993^b^	Metropolitan, *N* = 16,219^b^	Micropolitan, *N* = 5,712^b^	Small town, *N* = 1,520^b^	Rural, *N* = 5,542^b^
**Race**^a^					
White	24,694 (85%)	12,913 (80%)	5,149 (90%)	1,419 (93%)	5,213 (94%)
Black	1,101 (3.8%)	791 (4.9%)	188 (3.3%)	44 (2.9%)	78 (1.4%)
Hispanic	1,162 (4.0%)	869 (5.4%)	177 (3.1%)	18 (1.2%)	98 (1.8%)
AIAN	112 (0.4%)	17 (0.1%)	16 (0.3%)	7 (0.5%)	72 (1.3%)
Other	1,924 (6.6%)	1,629 (10%)	182 (3.2%)	32 (2.1%)	81 (1.5%)
**% on Medicare**	5.5 (3.9, 7.5)	5.0 (3.7, 6.7)	6.0 (4.4, 7.9)	6.7 (4.6, 9.6)	6.4 (4.4, 9.1)
**% on Medicaid**	12 (7, 19)	11 (6, 18)	15 (10, 20)	15 (9, 21)	13 (7, 19)
**% Uninsured**	7 (4, 11)	6 (4, 11)	7 (4, 12)	7 (4, 13)	7 (4, 12)
**Socioeconomic status**					
higher	24,631 (85%)	14,636 (90%)	4,611 (81%)	1,078 (71%)	4,306 (78%)
lower	4,362 (15%)	1,583 (9.8%)	1,101 (19%)	442 (29%)	1,236 (22%)

^a^Number of ZIP codes by predominant ethnicity/race

^b^n (%); Median (IQR)

The overall, average number of DEA-waivered prescribers per 100,000 was 19 (Table 2). Rates of DEA-waivered prescribers were higher in metropolitan (20 per 100,000 residents) and micropolitan areas (14 per 100,000 residents) compared to small town (7 per 100,000 residents) and rural areas (9 per 100,000 residents). Overall, predominantly AI/AN neighborhoods had the highest rate of DEA-waivered prescribers per 100,000 residents, followed by predominantly ‘Other’ neighborhoods, predominantly NHW neighborhoods, predominantly Black/African American neighborhoods and lastly, predominantly Hispanic/Latinx neighborhoods. These rates differed by neighborhood type, where the ordering of rates by majority racial group changed (Table 2).

**Table 2:** Average number of DEA-waivered prescribers per 100,000 by predominant race/ethnicity and rurality

	**Overall**^*a*^	**Metropolitan**^*a*^	**Micropolitan**^*a*^	**Small town**^*a*^	**Rural**^*a*^
White	19.4 (76.2)	21.0 (66.7)	14.6 (42.3)	7.0 (50.9)	8.7 (40.6)
Black	19.1 (67.7)	20.2 (61.5)	7.3 (17.7)	1.1 (7.5)	3.7 (15.0)
Hispanic	12.4 (34.9)	12.7 (31.5)	7.4 (19.8)	16.9 (52.0)	5.8 (22.1)
AIAN	23.9 (66.2)	4.7 (10.4)	30.6 (60.4)	47.9 (98.3)	24.7 (67.8)
Other	20.4 (53.8)	20.7 (51.5)	14.2 (28.9)	2.7 (8.6)	14.4 (40.8)
Overall	18.8 (71.3)	20.0 (61.4)	14.0 (40.2)	7.0 (49.5)	8.9 (40.9)

^*a*^Mean (SD)

**Fig. 1:**
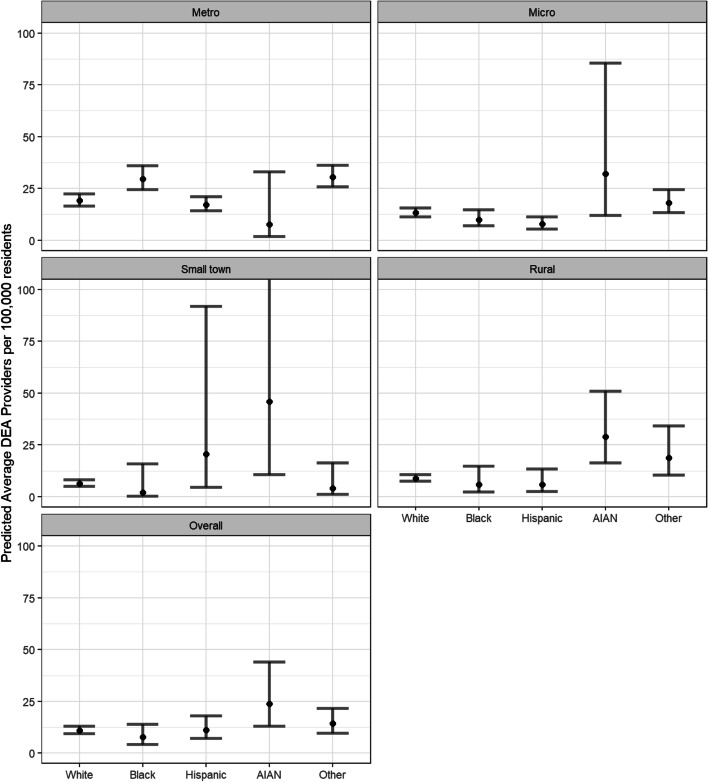
Model prediction of number of prescribers by racial/ethnic neighborhood zip codes and rurality, after setting covariate levels to: socioeconomic status (i.e., area deprivation index = most-deprived), percentage on Medicare = 6.29%, percentage on Medicaid = 14.18%, percentage uninsured = 8.37%. Metro indicates metropolitan and micro indicates micropolitan neighborhoods. Racial and ethnic categories are based on if the neighborhood is composed of > 50% of one race/ethnicity, then it is labeled according to that racial and ethnic group. Overall racial and ethnic group predictions are marginal mean estimates based on the fitted interaction model. The error bar for AI/AN in the small town group extends to 200, but was cutoff at 100 for readability

Analytically, we found there was an interaction between neighborhood type and predominant racial group in our fitted negative binomial model (χ^2^ = 41.5, *p* < 0.0001). The interaction is also apparent in the estimated rates derived from the model, adjusted for neighborhood SES, percent on Medicare, percent on Medicaid, and percent uninsured (Fig. 1). Figure 1 shows availability of DEA-waivered prescribers is highest for predominantly AI/AN neighborhoods in Micropolitan, Small town, and rural neighborhoods, but lowest for predominantly AI/AN neighborhoods in metropolitan areas.

Based on our Tukey adjusted comparisons of racial group within metropolitan areas, we found that predominantly NHW ZIP codes had a lower rate of DEA-waivered prescribers compared to predominantly Black/African American ZIP codes (RR = 0.65, CI = (0.54, 0.78). Also, within metropolitan areas, predominantly Hispanic/Latinx ZIP codes had lower rates of DEA-waivered prescribers available compared to predominantly Black/African American neighborhoods (RR = 0.58, CI = (0.46, 0.74)). Within micropolitan neighborhoods, predominantly Hispanic/Latinx ZIP codes had lower rates of DEA-waivered prescribers availability compared to predominantly NHW neighborhoods (RR = 0.59, CI = (0.37, 0.93)).

No definitive differences within small town ZIP codes could be determined, although predominantly AI/AN ZIP codes had the highest estimated average DEA-waivered prescribers per 100,000 residents. Within rural ZIP codes, predominantly AI/AN neighborhoods had higher DEA-waivered prescriber availability compared to predominantly White neighborhoods (RR = 3.23, CI = (1.49, 7.14)), compared to predominantly Black/African American neighborhoods (RR = 5.0, CI = (1.14, 20.0)), and compared to predominantly Hispanic/Latinx neighborhoods (RR = 5.0, CI = (1.28, 20.0)). All comparison can be found in the supplement (Table S1). DEA-waivered prescriber availability was lower for high SES Neighborhoods (RR = 0.68, CI = (0.63, 0.74)). A 10% increase in a neighborhood’s Medicare population, Medicaid population and those uninsured was associated with a 39% decrease (RR = 0.61, CI = (0.55, 0.67)), a 4% decrease (RR = 0.96, CI = (0.92, 0.99)), and a 21% decrease (RR = 0.79, CI = (0.74, 0.84)) in the average number of DEA-waivered prescriber, respectively. The District of Columbia has the highest number of DEA-waivered prescribers per capita, followed by Massachusetts and Rhode Island. Northeastern states in general are overrepresented among the highest levels of DEA-waivered prescribers (Figs. S1 and S2 are provided in the supplementary materials). The variance inflation factor for all model terms were less than 2, indicating no problems with multicollinearity.

## Discussion

In this study, we assessed availability of DEA-waivered buprenorphine prescribers across ZIP codes in the United States by population characteristics including race and ethnicity, SES, type of insurance, and urban–rural location. Unexpectedly, we observed that compared with predominantly NHW ZIP codes, some racially and ethnically diverse neighborhoods had a higher proportion of MOUD prescribers. The highest proportion of waivered prescribers were among predominantly AI/AN ZIP codes.In metropolitan areas, there were higher proportions of waivered prescribers in majority Black/African American ZIP codes. The exception to these findings were the reduced proportion of prescribers among primarily Hispanic/Latinx communities. Additional research is needed to determine the complex factors related to MOUD access among diverse communities which is vital to advancing health equity. This is especially critical following the rise in opioid- and fentanyl- related overdoses during the COVID-19 pandemic [35].

Our findings are supported by previous research that assessed potential access to prescribers among AI/AN communities, with results indicating there were a similar number of prescribers in AI/AN communities as in non-AI/AN areas [36]. Likewise, a recent study using a national treatment dataset determined that facilities serving AI/AN adults offered some medications at similar rates as facilities serving non-AI/AN clients, but were less likely to deliver standard treatment (i.e., methadone, buprenorphine; 22.4% vs 27.6%, *p*< 0.001) [37]. The relatively high availability of waivered prescribers may be the result of concerted efforts by Indian Health Service and other funding agencies such as the Substance Abuse and Mental Health Agency to improve treatment for this patient population.

Tribal nations have also worked to increase access to MOUD. For instance, the Swinomish Indian Tribal Community has reduced opioid fatalities among Tribal members by over 50.0% [38]. Despite the potential availability of prescribers, other factors related to health equity may be contributing to the disparity in fatal overdose among AI/AN adults. This may include the greater number of AI/AN adults living in urban areas which might introduce other barriers to care if services are primarily offered within the reservation community [39, 40]. As highlighted in Figure S2, it is also likely that although waivered prescribers are in rural areas, they may not be physically accessible to the individual in need of treatment [40].

There are also considerations in utilization of MOUD among Black/African American and Hispanic/Latinx adults. Although results from this study pointed to increased numbers of waivered prescribers in metropolitan Black/African American neighborhoods, there are challenges to receiving treatment such as fear of legal consequences [41] and a lack of culturally responsive care [11]. Counter to these findings, across regions, there were decreased waivered prescribers in predominantly Hispanic/Latinx neighborhoods. A recent report about the growing OUD crisis among Hispanic/Latinx adults highlighted the unique hurdles faced in these communities in obtaining treatment. Barriers to MOUD included concerns around impact on immigration status and a lack of prescribers that speak Spanish, Portuguese, or Indigenous languages [42]. Due to a financial or insurance reason, over 20% of Hispanic/Latinx adults also indicated that they experienced obstacles to accessing a usual source of care (e.g., a primary care provider), which may further decrease the likelihood of receiving office-based MOUD.

While more research is needed into why diverse communities may not be accessing waivered prescribers in their area, studies have also shown that type of OUD medication available to adults differs along racial lines which may impact access and points to structural racism [43, 44]. For example, among Black/African American and Hispanic/Latinx communities across the United States, methadone was more likely to be accessible than buprenorphine [45]. For patients where both treatments are effective, buprenorphine should be made available [43]. Buprenorphine is commonly viewed as preferable because it is less stigmatizing (i.e., accessible in primary care or office-based settings) and less constraining than receiving care at a methadone clinic which generally have highly restrictive rules around treatment [43, 44].

At the prescriber level, research has indicated that financial, reimbursement and insurance concerns, hesitation around training or prescribing, or state requirements around prescribing and prescribing in general are all obstacles to becoming waivered [23]. Previous research has also noted the need to address prescriber attitudes to increase interest in becoming waivered in rural regions [21, 46]. More than half  of prescribers located in rural areas that participated in a recent survey indicated that they had concerns around diversion or misuse of medication and approximately  a third did not feel like they had the ability to manage opioid use disorder, or lacked the specialty training to manage complex problems [21]. Counter to what one might expect, some prescriber training requirements have been removed in the hopes that this will motivate more prescribers to become waivered [47].

At the patient level, stigma, type of insurance (or lack of insurance), and location of prescribers may negatively impact access [48]. Practical solutions have been implemented. For example, mobile buprenorphine clinics have had promising pilot findings for treatment retention among Black/African American, Hispanic/Latinx and NHW veterans experiencing homelessness [23, 49]. More efforts are needed to increase the number of waivered prescribers or determine the appropriate number of waivered prescribers per 100,000 people as well as decrease patient barriers to MOUD to address the opioid overdose epidemic.

With respect to insurance and urban–rural region, our findings are in alignment with previous research that has noted the association between Medicaid insurance and decreased buprenorphine prescriptions as well as lower proportion of available prescribers due to rurality [17, 19, 50]. Compared to Metropolitan neighborhoods, we found that as regions become more rural, the average number of registered MOUD prescribers correspondingly decreased. Our results also suggest that Northeastern states had the highest proportion of buprenorphine prescribers in the nation. While the number of practitioners able to prescribe MOUD has been growing across the United States, it is still necessary to expand access in rural and lower-resourced areas and support practitioners in increasing buprenorphine prescriptions.

Policy plays a key role in the effort to improve access, with a recent study recommending more funneling of federal funds to existing healthcare mechanisms (e.g., Medicaid, Veterans Affairs, Indian Health Service) instead of focusing on time-limited grants that do not build the required treatment infrastructure. Additionally, the authors of the same study proposed enforcement of insurance parity laws across all insurance plans which would also assist with increasing the use of evidence-based treatment [51]. Telemedicine has been another strategy to increase access in rural areas and has been successful in expanding reach of buprenorphine [52]. Since the COVID-19 national public health emergency was declared in March of 2020, federal and state regulations around the delivery of buprenorphine through telemedicine have been relaxed in an effort to maintain access to MOUD. The potential for long-term adoption and sustainment of these changes in regulations have been encouraging.

There are limitations of our research that are important to note. Not all prescribers that have a waiver actively prescribe buprenorphine, with recent research indicating that only half of waivered practitioners prescribed buprenorphine over a two-year period [53]. We were also not able to accurately determine the number of active and inactive prescribers and our analysis did not specifically include methadone clinics. Still, our analysis accounted for practitioners prescribing in settings that are not typically reported in the literature (e.g., inpatient and outpatient hospital settings, detention, and mental health centers). This study included US Census Bureau RUCA categories that incorporate population density and commuting patterns to measure availability of prescribers but does not consider wait time or other factors that may influence actual access or ability to use services. Defining areas by the predominant racial and ethnic group may obscure other racial and ethnic populations differences in access to buprenorphine. Also, by controlling for SES and relative deprivation, which are social determinants of health, the need of racial and ethnic groups may be underestimated, such as factors related to economic opportunity and education.

We do not have the estimates for the number of people that might want MOUD services per ZIP code. Additional research is therefore warranted that includes both prescriber and population data to better determine the need for MOUD. More research is also necessary into the treatment needs of Hispanic/Latinx adults and what barriers may exist for these communities. Additionally, there are limitations in the use of the American Community Survey which is conducted by the US Census Bureau. Misclassification of racial and ethnic populations in Census data is prevalent, although steps are taken to validate the demographic information collected by the American Community Survey. Also, the small number of ZIP codes that were predominantly racially and ethnically diverse may have prevented detection of statistically significant differences, should they exist. Lastly, we did not examine spatial autocorrelation and future research may include this to better understand spatial patterns. However, the strength of this research is in our use of The Drug Enforcement Administration Registration Controlled Substances Act Information Database which allows for a more exact and granular assessment of practitioners waivered to prescribe buprenorphine across the United States.

## Conclusions

This analysis of the DEA Registration controlled Substances Act Information Database revealed higher proportions of waivered prescribers for AI/AN and ‘Other’ ZIP codes. In metropolitan areas, Black/African American people had a greater proportion of prescribers than NHWs, with Hispanic/Latinx adults experiencing low access. As expected, rural neighborhoods had lower proportions of waivered prescribers. With the continuing opioid overdose epidemic, more solutions such as increased numbers of waivered prescribers and policy changes are needed. Future research should address the delineation of active versus inactive waivered prescribers. In addition, further examination is necessary into the discrepancy between the higher proportion of waivered prescribers among diverse communities but increased recent fatality rates among AI/AN and Black/African American populations. Cultural adaptations of MOUD to increase uptake among diverse populations are also necessary, as are ways to decrease patient level barriers to MOUD to enhance health equity.

## Supplementary Information

**Additional file 1: Figure S1.** State differences in prescriber availability. States are first ordered by median number of providers per 100,000 people, then ordered by the upper quartile number of providers per 100,000. **Figure S2.** Number of DEA prescribers per 100,000 people in zip codes across the U.S.

**Additional file 2: Table S1.** Pairwise comparison of predominant race/ethnicity group within rurality type. Estimates were derived from the fitted negative binomial model with random intercepts for state and adjusted for neighborhood deprivation, percent on Medicaid, percent on Medicare, and percent uninsured at the ZIP code level.

## Data Availability

The data that support the findings of this study are available from The Drug Enforcement Administration Registration Controlled Substances Act Information Database, but restrictions apply to the availability of these data, which were used with approval for the current study, and so are not publicly available. Data are however available from the authors upon reasonable request and with permission of The Drug Enforcement Administration Registration Controlled Substances Act Information Database. Data were also obtained from the U.S. Census Bureau, American Community Survey 5-Year Estimates, available at https://www.data.census.gov/.
