# Supplementary material for: Variations in national availability of waivered buprenorphine prescribers by racial and ethnic composition of zip codes
Source: Subst Abuse Treat Prev Policy. 2022 May 25;17:41. doi: 10.1186/s13011-022-00457-3 (PMC9131568; doi:10.1186/s13011-022-00457-3)

**Figure S1.**State differences in prescriber availability. States are first ordered by median number of providers per 100,000 people, then ordered by the upper quartile number of providers per 100,000.


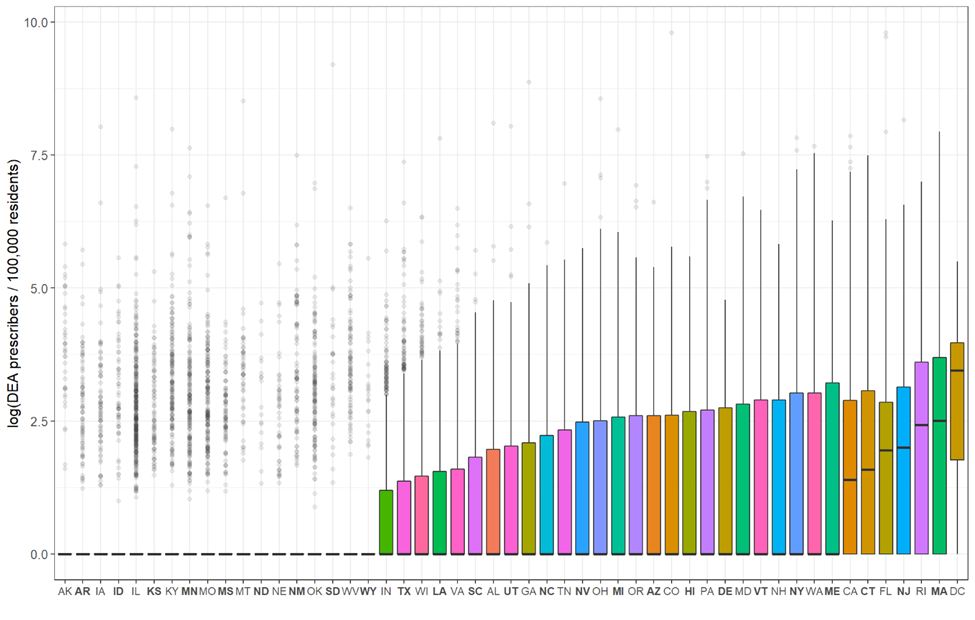
 **Figure S2.** Number of DEA prescribers per 100,000 people in zip codes across the U.S
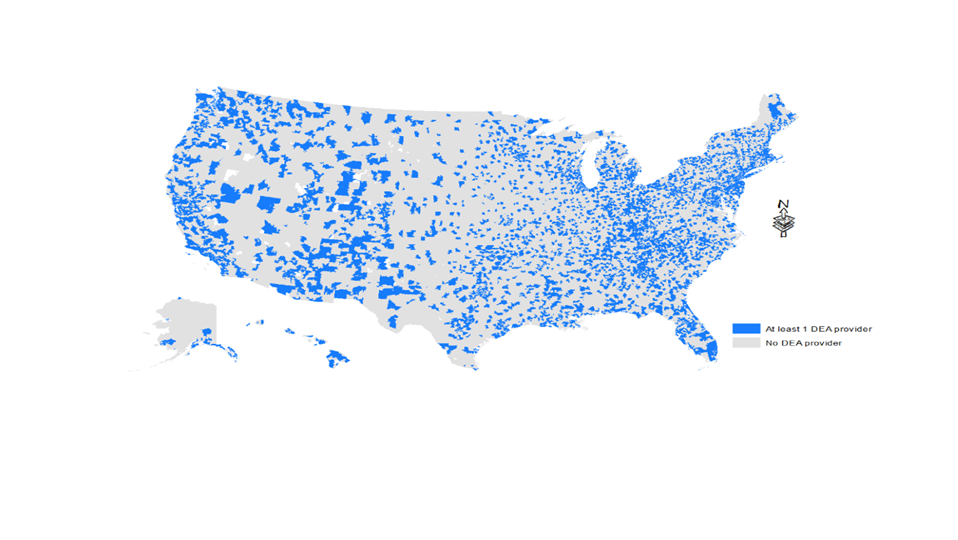

Supplement: Supplementary file 1 — Additional file 1: Figure S1. State differences in prescriber availability. States are first ordered by median number of providers per 100,000 people, then ordered by the upper quartile number of providers per 100,000. Figure S2. Number of DEA prescribers per 100,000 people in zip codes across the U.S. [file 13011_2022_457_MOESM1_ESM.docx]
