# Supplementary material for: Variations in national availability of waivered buprenorphine prescribers by racial and ethnic composition of zip codes
Source: Subst Abuse Treat Prev Policy. 2022 May 25;17:41. doi: 10.1186/s13011-022-00457-3 (PMC9131568; doi:10.1186/s13011-022-00457-3)
Supplement: Supplementary file 2 — Additional file 2: Table S1. Pairwise comparison of predominant race/ethnicity group within rurality type. Estimates were derived from the fitted negative binomial model with random intercepts for state and adjusted for neighborhood deprivation, percent on Medicaid, percent on Medicare, and percent uninsured at the ZIP code level. [file 13011_2022_457_MOESM2_ESM.docx]

| **contrast** | **rurality** | **RR***^1^* | **CI** | **p***^2^* |
| --- | --- | --- | --- | --- |
| White / Black | Metro | 0.65 | (0.54, 0.78) | <0.001 |
| White / Hispanic | Metro | 1.12 | (0.92, 1.35) | 0.532 |
| White / AIAN | Metro | 2.52 | (0.33, 19.18) | 0.726 |
| White / Other | Metro | 0.63 | (0.56, 0.72) | <0.001 |
| Black / Hispanic | Metro | 1.72 | (1.36, 2.18) | <0.001 |
| Black / AIAN | Metro | 3.89 | (0.51, 29.71) | 0.360 |
| Black / Other | Metro | 0.98 | (0.8, 1.19) | 0.997 |
| Hispanic / AIAN | Metro | 2.26 | (0.3, 17.2) | 0.808 |
| Hispanic / Other | Metro | 0.57 | (0.47, 0.69) | <0.001 |
| AIAN / Other | Metro | 0.25 | (0.03, 1.91) | 0.339 |
| White / Black | Micro | 1.33 | (0.81, 2.18) | 0.504 |
| White / Hispanic | Micro | 1.70 | (1.07, 2.71) | 0.016 |
| White / AIAN | Micro | 0.42 | (0.11, 1.62) | 0.400 |
| White / Other | Micro | 0.74 | (0.5, 1.08) | 0.187 |
| Black / Hispanic | Micro | 1.28 | (0.66, 2.49) | 0.853 |
| Black / AIAN | Micro | 0.31 | (0.07, 1.32) | 0.178 |
| Black / Other | Micro | 0.55 | (0.3, 1.01) | 0.059 |
| Hispanic / AIAN | Micro | 0.24 | (0.06, 1.02) | 0.054 |
| Hispanic / Other | Micro | 0.43 | (0.24, 0.78) | <0.001 |
| AIAN / Other | Micro | 1.77 | (0.44, 7.19) | 0.801 |
| White / Black | Small town | 3.17 | (0.18, 55.95) | 0.810 |
| White / Hispanic | Small town | 0.31 | (0.04, 2.49) | 0.539 |
| White / AIAN | Small town | 0.14 | (0.02, 1.07) | 0.065 |
| White / Other | Small town | 1.53 | (0.23, 10.32) | 0.975 |
| Black / Hispanic | Small town | 0.10 | (0.003, 3.32) | 0.374 |
| Black / AIAN | Small town | 0.04 | (0.001, 1.46) | 0.106 |
| Black / Other | Small town | 0.48 | (0.02, 14.8) | 0.978 |
| Hispanic / AIAN | Small town | 0.45 | (0.02, 8.12) | 0.944 |
| Hispanic / Other | Small town | 4.94 | (0.3, 81.3) | 0.527 |
| AIAN / Other | Small town | 10.96 | (0.69, 174.4) | 0.126 |
| White / Black | Rural | 1.53 | (0.43, 5.45) | 0.895 |
| White / Hispanic | Rural | 1.53 | (0.49, 4.82) | 0.851 |
| White / AIAN | Rural | 0.31 | (0.14, 0.67) | <0.001 |
| White / Other | Rural | 0.47 | (0.21, 1.06) | 0.086 |
| Black / Hispanic | Rural | 1.00 | (0.18, 5.49) | >0.999 |
| Black / AIAN | Rural | 0.20 | (0.05, 0.88) | 0.026 |
| Black / Other | Rural | 0.31 | (0.07, 1.38) | 0.202 |
| Hispanic / AIAN | Rural | 0.20 | (0.05, 0.78) | 0.011 |
| Hispanic / Other | Rural | 0.31 | (0.08, 1.24) | 0.141 |
| AIAN / Other | Rural | 1.53 | (0.51, 4.61) | 0.829 |
| *^1^*RR = Rate ratio  *^2^*P-values were adjusted via Tukey's method for multiple comparisons. | | | | |

**Table S1.** Pairwise comparison of predominant race/ethnicity group within rurality type. Estimates were derived from the fitted negative binomial model with random intercepts for state and adjusted for neighborhood deprivation, percent on Medicaid, percent on Medicare, and percent uninsured at the ZIP code level.
